# Immunolocalization of *Beet Curly Top Virus* (BCTV) and GroEL Chaperon Protein of Endosymbionts in Beet Leafhopper (*Circulifer tenellus*) Vector Tissue

**DOI:** 10.3390/v16101571

**Published:** 2024-10-05

**Authors:** Batool M. Alkhatib, Samy Belteton, Rebecca Creamer

**Affiliations:** 1 Molecular Biology and Interdisciplinary Life Sciences, New Mexico State University, Las Cruces, NM 88003, USA; 2 Microscopic Imaging Facility, New Mexico State University, Las Cruces, NM 88003, USA; sbelteto@nmsu.edu; 3 Department of Entomology, Plant Pathology, and Weed Science, New Mexico State University, Las Cruces, NM 88003, USA

**Keywords:** *geminiviridae*, beet curly top virus, *Circulifer tenellus*, endosymbionts, GroEL immunofluorescence, confocal laser scanning microscopy

## Abstract

Beet curly top virus (BCTV, *curtovirus*, *geminiviridae*) causes one of the most economically significant viral diseases in crops in the Western United States and is transmitted only by the beet leafhopper (*Circulifer tenellus*) in a non-propagative circulative manner. A better understanding of how this virus overcomes insect vector cellular barriers is essential to understanding virus–vector interactions. The distribution of BCTV in its beet leafhopper vector was investigated using immunofluorescence confocal laser scanning microscope analysis (iCLSM) on the whole-mount-dissected organs of leafhoppers. BCTV was localized in several lobes of the principal salivary glands, filter chamber, anterior midgut, and mid midgut, suggesting the occurrence of midgut and salivary gland barriers to BCTV transmission in its vector *C. tenellus*. This study also investigated the distribution of the chaperon GroEL homolog protein produced by primary endosymbiotic bacteria within the beet leafhopper, which is believed to indirectly affect viral transmission by enhancing insect immunity and resistance to viruses. GroEL was identified in leafhopper salivary glands lobes, the stylet, salivary canal, the filter chamber, and the Malpighian tubule. This is the first work to visualize the localization of a *curtovirus* within its beet leafhopper vector. Together, these results can help understand ssDNA virus–vector relationships, including cellular transmission barriers and other vector protein components.

## 1. Introduction

Insect-transmitted plant viruses encompass several species, some of which have emerged or re-emerged as significant challenges to crop cultivation [1,2,3]. Within the approximately 1200 insect-vectored plant viruses, only two families possess a single-stranded DNA (ssDNA) genome: *geminiviridae* and *Nanoviridae*. All viruses within these families rely on phloem-feeding insects for transmission.

The majority of species within these families are recognized for their circulative non-propagative transmission mode [4]. In this transmission mode, the ingested virus is internalized and circulates through the vector’s body until it is eventually excreted with the saliva without replicating during this process. For successful virus transmission, the virus must cross physiological barriers, mainly the midgut, hemolymph, and salivary glands. The most studied model among the geminiviruses in terms of localization and interaction with insect vectors is the begomovirus pathway of the tomato yellow leaf curl virus (TYLCV) through *Bemisia tabaci* whitefly species [5,6,7]. The specific barriers to begomovirus transmission include acquisition through plant sap, movement through the midgut, entry to the hemolymph, and subsequently, movement into the primary salivary glands (PSGs) from which they are eventually secreted in the saliva [7,8,9]. The geminivirus capsid protein (CP) is the major and likely only determinant of the viral cycle inside the insect. The CP is located at the surface of the virion, where it is in contact with several insect tissues, from ingestion to egestion. As begomoviruses (family *geminiviridae*) move through their whitefly vector, the CP is the only virus protein exposed to whitefly tissues and available to interact with insect receptors or other proteins that help in or facilitate the translocation of the virus through insect tissues. In a recombinant chimera virus experiment, the exchange of the CP gene of the whitefly-transmissible *African cassava mosaic virus* with that of the leafhopper-transmitted *beet curly top virus* resulted in a leafhopper-transmissible variant [10]. Overall, the current data indicate that the key viral determinant of vector transmission is the CP of geminiviruses [8,10,11].

*Beet curly top virus* (genus *Curtovirus*; family *Geminiviridae)* is transmitted plant-to-plant, exclusively by the beet leafhopper (*Circulifer tenellus*). The beet leafhopper is an efficient vector of BCTV. The virus is acquired from an infected plant within seconds, and the feeding time necessary for the transmission of the virus to a new healthy plant can be as short as 1 min; however, longer feeding times (for example, inoculation access periods (IAP) of 24–48 h) provide higher rates of infection. Depending on the length of the acquisition access period (AAP), leafhoppers can remain viruliferous for life [12]. The pathway BCTV follows the beet leafhopper, which was investigated with a PCR-based approach; virus particles were detected in the digestive tract after an acquisition access period of 1 h, in the hemolymph after 3 h, and in the salivary glands after 4 h, with the saliva, the virus reintroduced into plants during feeding [12].

Insects frequently harbor bacterial endosymbionts that offer them nutritional support and/or protection against natural enemies, plant defenses, abiotic stresses, and insecticides. However, specific endosymbionts may also change insect vectors’ transmission and acquisition of plant pathogens [13]. Many leafhoppers harbor bacterial endosymbionts, significantly impacting the insects’ biology and behavior. Among these endosymbionts, the obligate ‘Candidatus *Sulcia muelleri’* is harbored by all leafhoppers, residing within a specialized organ called the bacteriome with the synthesis of at least eight essential amino acids that are lacking in the insects’ phloem sap diet [14,15].

Candidatus *Sulcia muelleri* is a primary endosymbiont present in the beet leafhopper, the GroEL housekeeping gene identified from the *Ca. S. muelleri* endosymbiont differs from all other strains published in NCBI, suggesting a new strain named *S. muelleri* beet leafhopper (SMBLH) [16]. The analysis of nucleotide and translated sequences, using alignment, phylogenetic trees, and predicted secondary and tertiary structures of a GroEL-homolog protein encoded by GroEL (GroHp), showed that it shares conserved regions and ATP binding sites from *Buchnera*, *Hamiltonella*, and *E. coli* GroHps [16].

The whitefly *Bemisia tabaci* has endosymbiotic bacteria in their bacteriocytes, which have a key role in protecting begomoviruses inside the hemolymph of the whitefly. By using an overlay assay, they confirmed the interaction between GroEL encoded by a secondary endosymbiont in *B. tabaci* and TYLCV particles [17]. Additionally, the coat protein of TYLCV and GroEL of *B. tabaci* showed a physical interaction in the yeast two-hybrid assay [18]. Feeding *B. tabaci* whiteflies with an antibody against its GroEL significantly decreased the efficiency of TYLCV transmission [17].

Despite the significant importance of the impact of ssDNA viruses in developing countries worldwide [19,20], the means by which they are transmitted remain primarily unknown since most studies focused on elucidating the transmission mechanisms of RNA viruses; previous studies on ssDNA viruses also focused on the role of viral proteins during transmission [21]. In contrast, the genetic and physiological mechanisms of the vectors responsible for viral transmission have remained mainly unexplored because dissecting insects, particularly phloem-feeding insects, is significantly challenging [22].

In the current work, we investigated the localization of an agriculturally and economically significant member in the geminiviruses family, the *beet curly top virus*. We used an immunofluorescence confocal laser microscope to examine the distribution of BCTV within its sole vector, the beet leafhopper. The same methodology was used to study the localization of the GroEL protein produced by *Ca. S. muelleri,* which is the primary endosymbiont of the beet leafhopper. This study should pave the way for further analysis of these essential bacteria since GroEl may enhance the insect’s capability to acquire or tolerate viruses in the beet leafhopper vectors and/or facilitate their transmission. These new findings expanded our knowledge of the interactions between virions and their insect vectors during plant virus persistent transmission.

## 2. Materials and Methods

### 2.1. Sources and Maintenance of Virus and Vector

The beet curly top virus severe strain (BCTV-Svr) was originally obtained from Carl Strausbaugh, USDA Kimberly, ID, USA, and was maintained on sugar beet (*Beta vulgaris*) using the beet leafhopper vector [BLH] (*Circulifer tenellus*). Insects were reared in a growth chamber with a 17 h photoperiod and held at 27 ± 2 °C during the light and 20 ± 2 °C during the dark periods. The leafhoppers and the sugar beets were tested frequently to confirm the BCTV-Svr infection using PCR with specific primers set (BCTV-CP4F, BCTV-CP6R) as previously reported [23]. Healthy (non-infected) beet leafhoppers were obtained from Punya Nachappa, Colorado State University, Fort Collins, CO, USA, and used as a control.

### 2.2. Whole Mount Preparation of Beet Leafhopper Organs for Immunofluorescence Confocal Laser Scanning Microscope (iCLSM)

Adult leafhoppers, both infected and control (non-infected), were collected by aspiration into small vials from infected sugar beets. Control and BCTV-infected adults were starved for 3–4 h to help clear plant material from the gut and were then processed for immunofluorescence, as mentioned below. After starvation, BLHs were immobilized by freezing at −20 °C for 10–15 min and then dissected on a microscope slide under a stereo microscope. Two fine forceps (Dumont # 5; Fine Science Tools (Foster City, CA, USA) Inc. no. 5) were cleaned with 70% ethanol and used for dissections.

Leafhoppers were dissected in 0.1 M phosphate-buffered saline (PBS), pH 7.4, under a stereo microscope. The head with attached salivary glands was gently pulled out from the thorax/abdomen using two fine forceps. Using one forceps, slight pressure was applied against the middle part of the cut abdomen, which pushed the filter chamber and gut out and away from other tissues; alternatively, the gut could be expelled by slightly tearing the cuticle and exposing the organs. All dissecting organs were quickly immersed in the fixative (4% paraformaldehyde in 0.1 M PBS, pH 7.4, 0.1% Triton X-100) in eight well chambers (ibidi USA, Inc., Fitchburg, WI, USA).

Three separate experiments were performed; each experiment was initiated using approximately 20 guts and 20 salivary glands.

### 2.3. Leafhopper Organs Used for Immunofluorescence

Fixed organs were processed for immunofluorescence, as described by [24,25], with some modifications. Fixed organs were washed in PBS (pH 7.4, 0.1% Triton X-100) (PBS-T) for 3 × 5 min, then permeabilized in PBS containing 1% Triton X-100 overnight at 4 °C. The next day, the organs were immersed in a blocking buffer (PBS-T—10% normal goat serum) for 30 min and then incubated for 3 h with a primary antibody (rabbit-produced) for either the BCTV coat protein (BCTV-CP) [26] or GroEL protein (Sigma-Aldrich, St. Louis, MO, USA) diluted at 1/300 in an incubation buffer (PBST—1% normal goat serum). They were washed in PBS-T for 3 × 5 min and then incubated for 2 h at room temperature with a 1/700 dilution of the goat anti-rabbit Alexa Fluor Plus 488 secondary antibody (Thermo Fisher, Waltham, MA, USA). 

Finally, samples were washed and mounted in propidium iodide (PI) containing Fluoro Gel III mounting media (Electron Microscopy Science, Hatfield, PA, USA). The PI-stained cell nuclei were red in contrast with the green fluorescence of Alexa Fluor 488. Samples were viewed under 20× and 40× and examined by the confocal Microscope Leica TCS SP5 II(Leica Microsystems, Deerfield, IL, USA). Starting with the secondary antibody step, the samples were protected from light as much as possible.

## 3. Results

### 3.1. BCTV Coat Protein Localization

**Figure 1:**
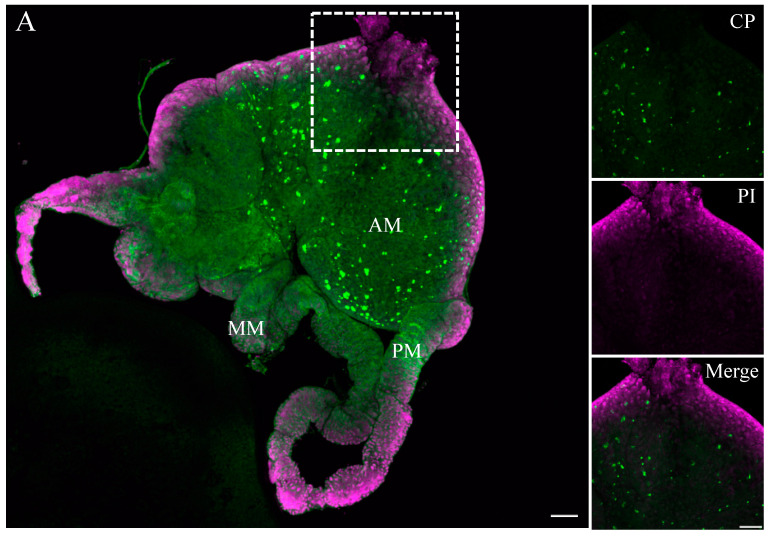

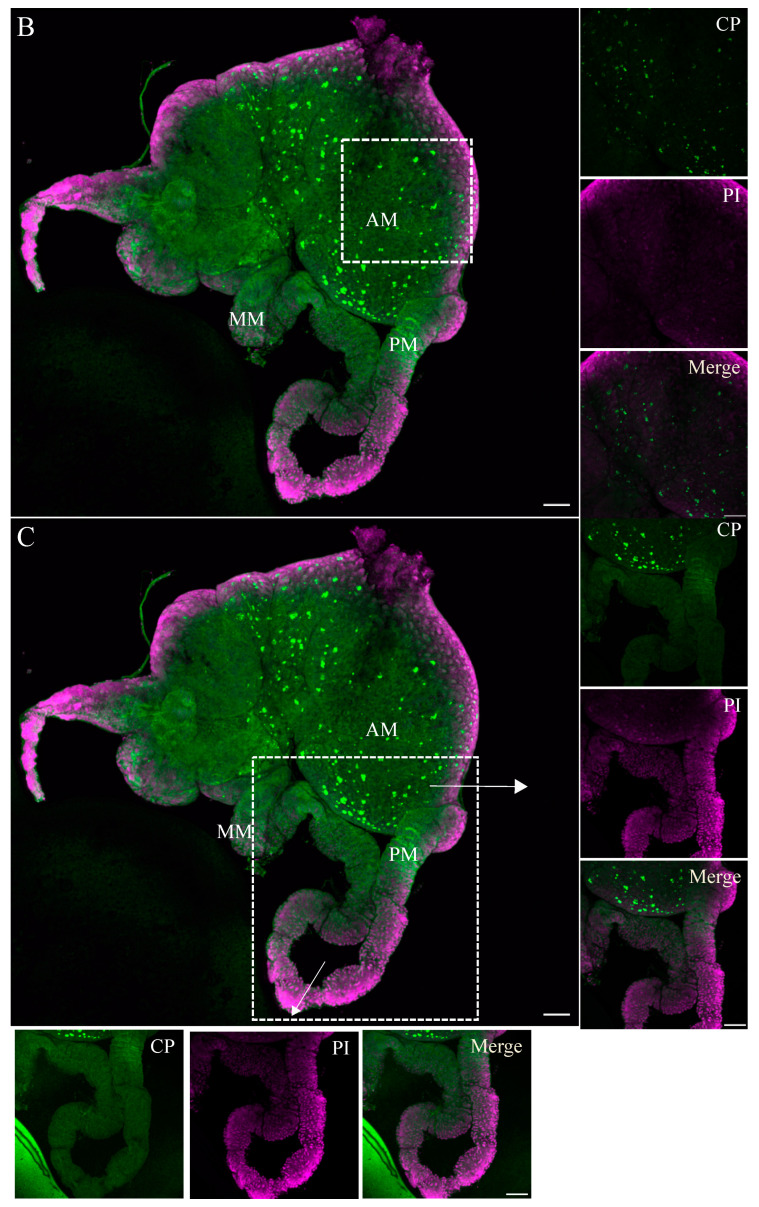
Confocal images of the whole-mount BCTV-infected midgut of the beet leafhopper (BLH) vector. BLH midguts were immunolabeled for BCTV-CP with Alexa Fluor (green), stained for nuclei with propidium iodide, PI (magenta), then examined by the confocal microscope (**A**–**C**), dissecting the midgut of infected BLH, and showing the anterior midgut (AM), mid midgut (MM), and the posterior midgut (PM). (**A**,**B**) BCTV-CP spread to more cells of the anterior midgut (AM) and (**C**) rarely to the other parts of the gut. The boxed region in each image is enlarged and shown in three different panels on the right side and bottom, representing a magnified view of different parts within the midgut. Scale bars = 100 μm.

**Figure 2:**
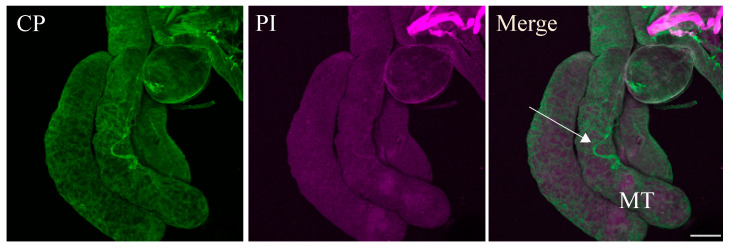
Immunofluorescence localization of beet curly top virus (BCTV) in the Malpighian tubule of beet leafhopper (BLH). Virus particles were localized using Alexa Fluor 488 (green), whereas cell nuclei were stained with propidium iodide PI (magenta). BCTV was detected in a few cells of the Malpighian tubule MT (arrow). MT; Malpighian tubule. Scale bar = 100 μm.

To study the distribution of beet curly top virus (BCTV) in its vector beet leafhopper (BLH), the morphology of the BLH was verified by the stereo and light microscope to ensure that the correct whole organ was dissected Specific green fluorescence, indicating the occurrence of BCTV coat protein (BCTV-CP) antigens, was detected with iCLSM in the whole-mount organs of BLH. Based on the morphology of BLH, the BCTV antigens were detected in the filter chamber and mainly in the anterior part of the midgut (Figure 1A–C). A weak specific immunofluorescence signal of the BCTV was observed in Malpighian tubules of BCTV-infected BLH (Figure 2).

**Figure 3:**
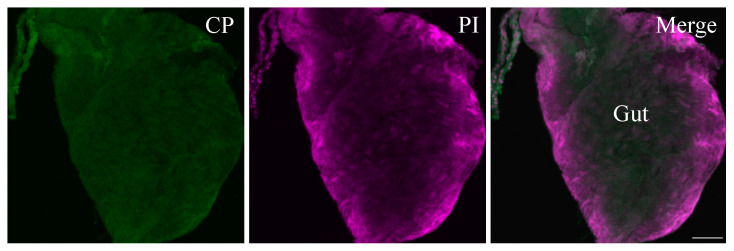
Immunofluorescence localization in the whole-mount midgut of a non-infected beet leafhopper (BLH). No signal was detected in the dissecting midgut incubated with Alexa Fluor Plus 488 (green). Nuclei were stained with propidium iodide (PI) magenta. Scale bar = 100 μm.

**Figure 4:**
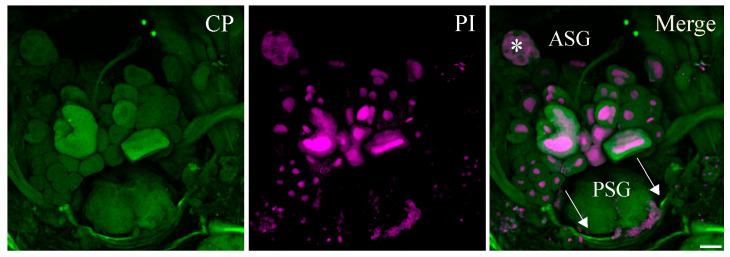
Confocal images of BCTV-infected salivary gland of the beet leafhopper (BLH) vector. Salivary glands of BLH were immunolabeled for BCTV with Alexa Fluor (green), stained for nuclei with propidium iodide PI (magenta), and then examined by the confocal microscope. BCTV is associated with the principal salivary glands PSGs (arrows). Asterisk: accessory salivary gland (ASG). Scale bar = 100 μm.

**Figure 5:**
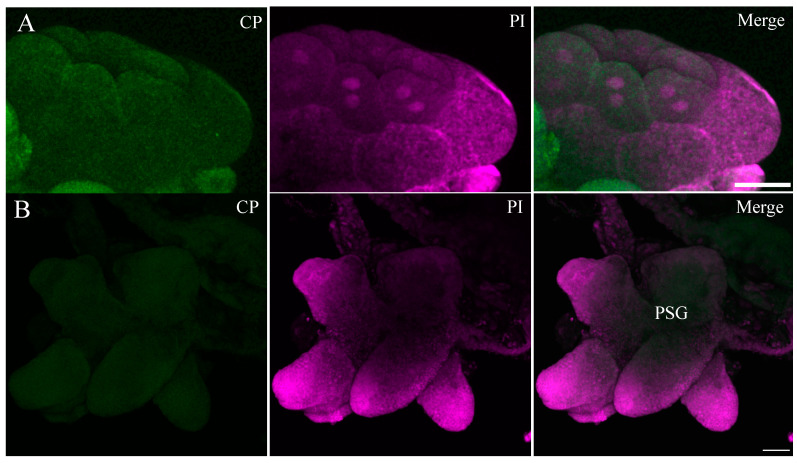
Confocal images of the non-infected salivary glands (negative control) of the beet leafhopper. Salivary glands were incubated with the goat anti-rabbit Alexa Fluor Plus 488. BCTV was not detected in any part of the different types of salivary gland cells; (**A**) types IV, V, and VI salivary cells, (**B**) types II and III salivary cells. Nuclei were stained with propidium iodide PI (magenta). Scale bar = 50 μm.

No BCTV-specific fluorescence was detected in whole-mount midguts from the negative control leafhoppers, which were not exposed to the virus (Figure 3). The salivary gland of the beet leafhopper consists of pairs of principal glands (PGs) and accessory glands (AGs); the PGs contain several lobes with different types of cells. Confocal images showed that BCTV was associated with the cell periphery in PSG (Figure 4). The negative control (non-infected) BLH showed no signal for BCTV in different parts of the salivary glands SGs (Figure 5A,B).

### 3.2. GroEL Protein of Primary Endosymbiont Localization

GroEL, which is indicative of endosymbiont presence, was detected by the analysis and dissection of over 25 salivary glands and guts of beet leafhoppers. Immunofluorescence localization analysis with a primary antibody specific for GroEL protein was used to reveal the distribution of GroEl within different organs of the beet leafhoppers.

**Figure 6:**
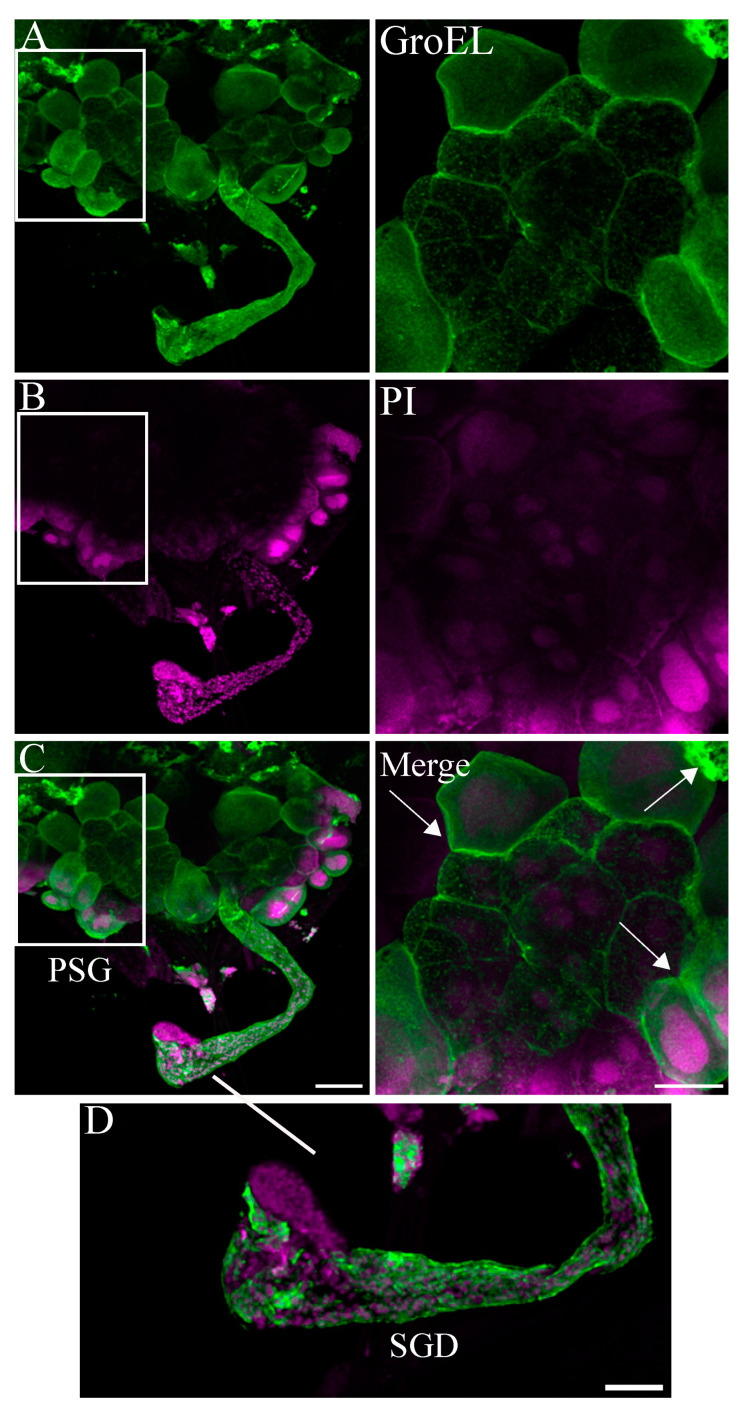
Immunofluorescence of GroEL of endosymbionts in salivary glands of beet leafhopper. (**A**–**C**) GroEL was detected in the salivary gland and (**D**) the salivary gland duct (SGD). (**A**–**C**) Scale bar = 100 μM; (**D**) = 50 μM. The boxed region in each image was enlarged and shown in three different panels on the right side, representing a magnified view of salivary gland lobes and showing the association of GroEL with the cell periphery in principle salivary glands (arrows). Scale bars = 50 μm. Principle salivary gland (PSG); salivary gland duct (SGD).

**Figure 7:**
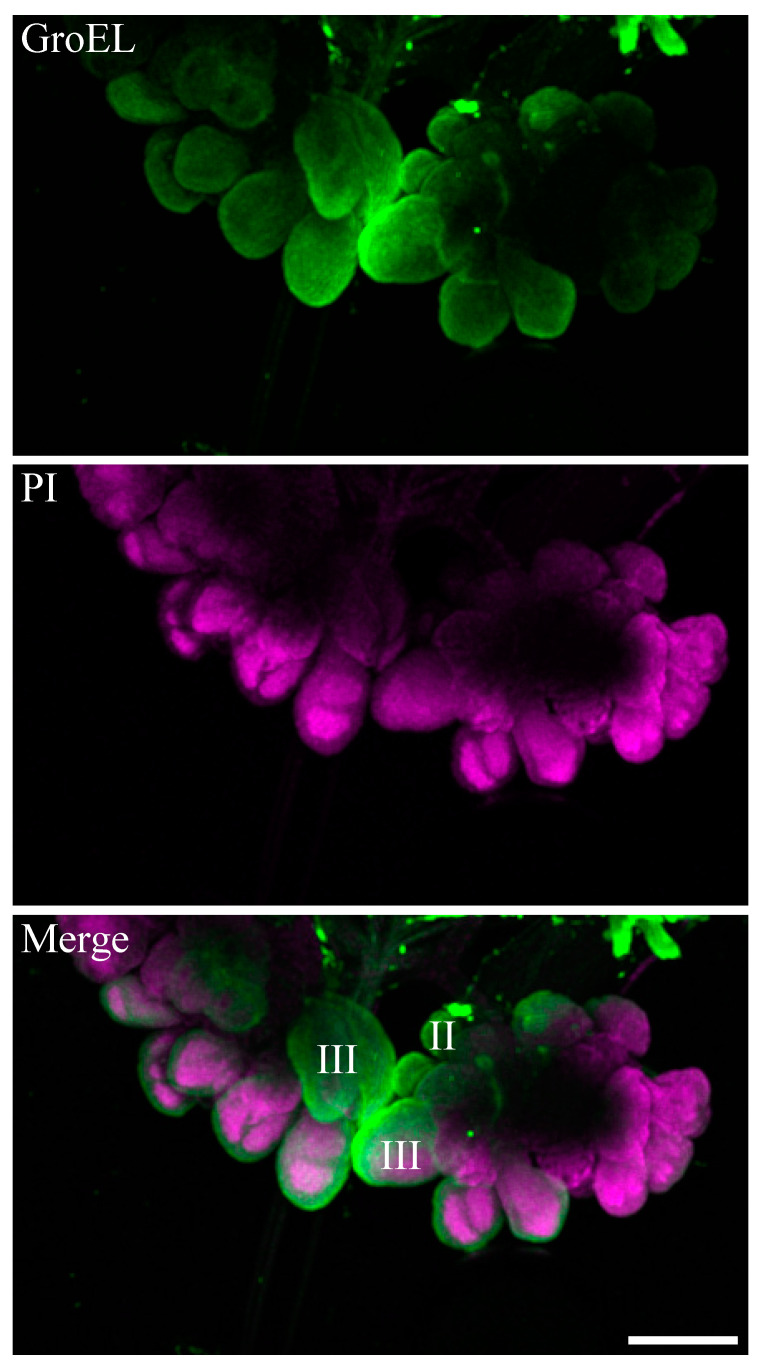
Immunofluorescence microscopy showing the distribution of GroEL in salivary glands of a viruliferous beet leafhopper. The GroEL protein was localized using Alexa Fluor 488 (green), whereas cell nuclei were stained with PI (magenta). Immunofluorescence microscopy showing the distribution of GroEL, mainly in type II and III principal salivary glands. Scale bar = 100 μM.

**Figure 8:**
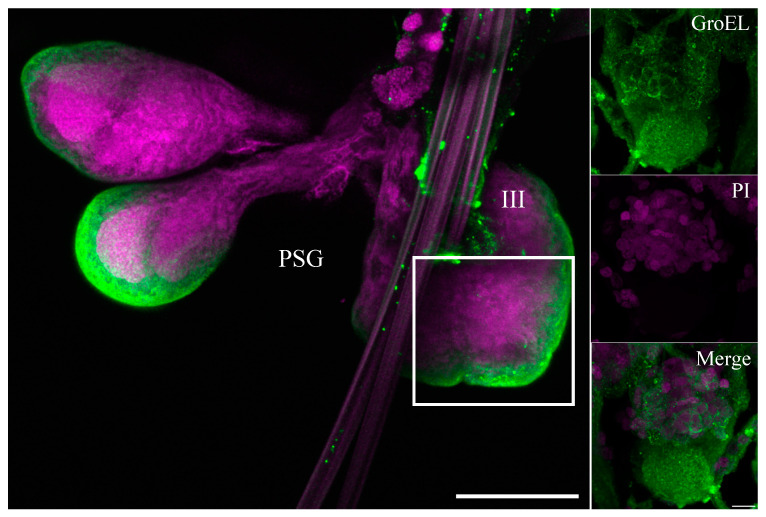
Immunofluorescence microscopy showing the distribution of GroEL in salivary gland lobes of a viruliferous beet leafhopper. The GroEL protein was localized using Alexa Fluor 488 (green), whereas cell nuclei were stained with PI (magenta). Immunofluorescence microscopy showed a large distribution of GroEL, mainly in type III cells of the principal salivary glands PSG. The boxed region is enlarged and shown in three panels on the right side, representing a magnified view of type III cells of the principal salivary gland. Scale bars = 10 μM.

**Figure 9:**
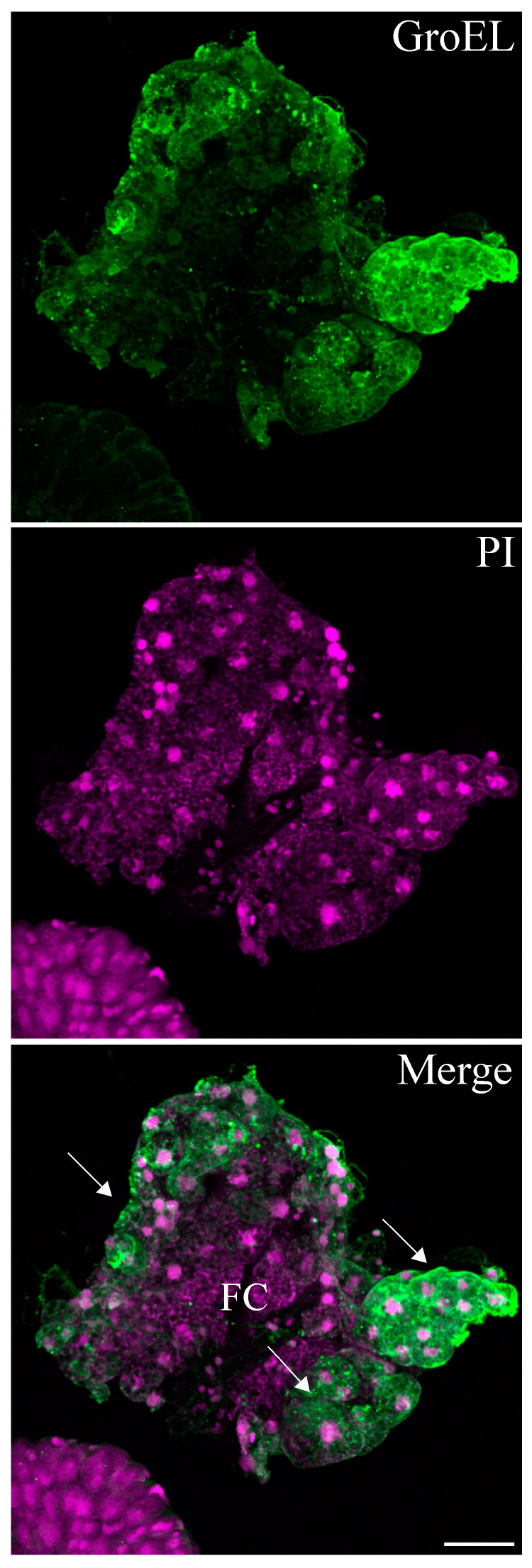
GroEL is associated with the filter chamber cells of the beet leafhopper. The GroEL protein was localized using Alexa Fluor 488 (green), whereas cell nuclei were stained with PI (magenta). The accumulation of the GroEL protein is shown (green fluorescence), as indicated by arrows, in the majority of the filter chamber (FC) cells dissected from the alimentary canal of the beet leafhopper. Scale bars = 50 μM.

**Figure 10:**
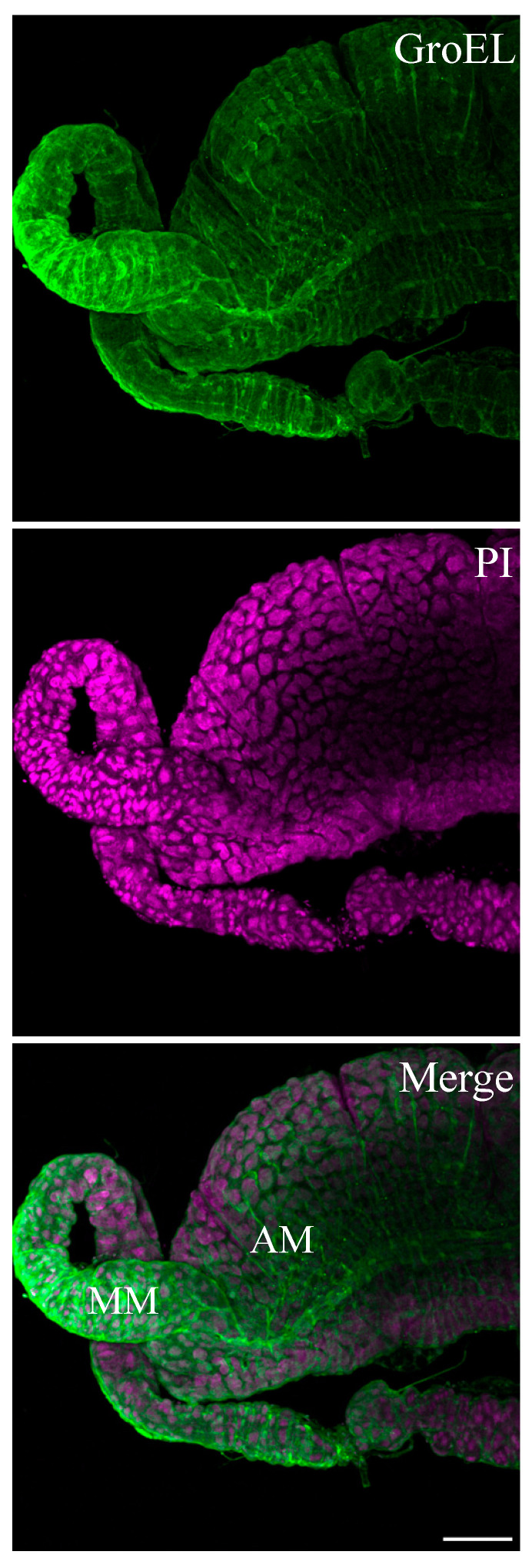
Localization of GroEL protein from primary endosymbionts in the beet leafhopper gut by immunofluorescence confocal microscopy. The GroEL protein was localized using Alexa Fluor 488 (green), whereas cell nuclei were stained with PI (magenta). The detection of the GroEL protein (green fluorescence) was mainly in the muscles of the anterior midgut (AM) and mid midgut (MM). Scale bar = 100 μM.

**Figure 11:**
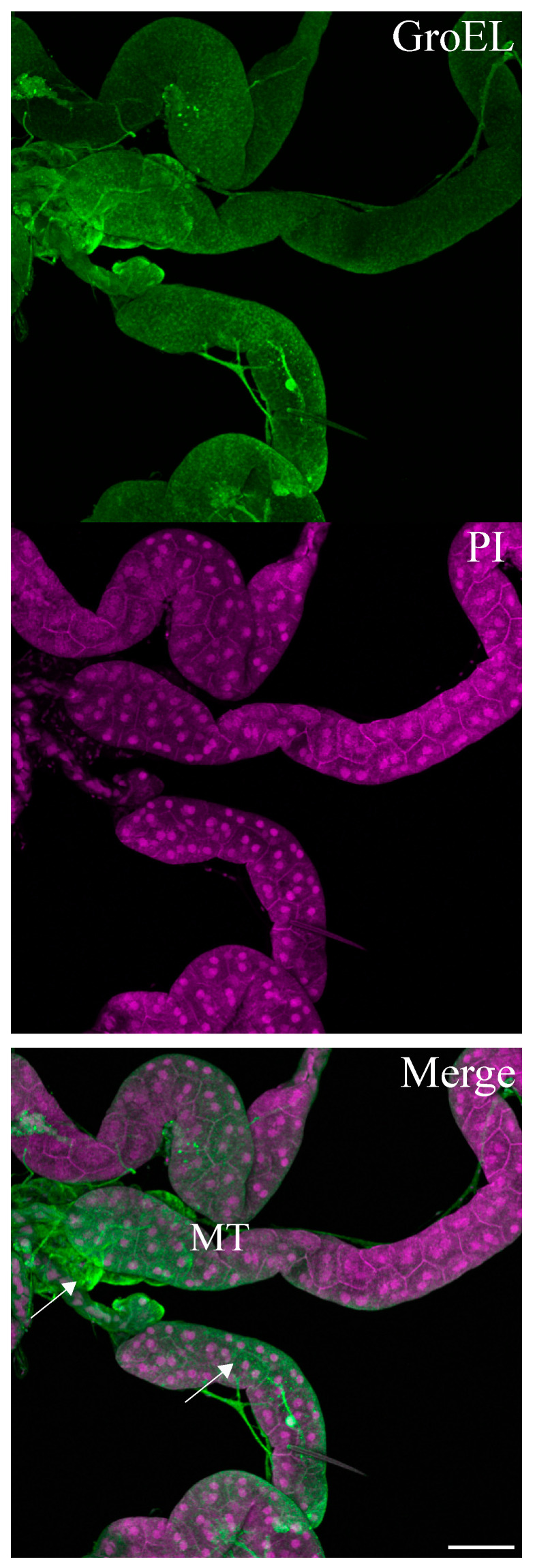
Immunofluorescence localization of the GroEL protein in Malpighian tubules of the beet leafhopper BLH. The GroEL protein was localized using Alexa Fluor 488 (green), whereas cell nuclei were stained with PI (magenta). GroEl is spread through the cells of different parts of the Malpighian tubule (MT arrow). Scale bar = 100 μM.

**Figure 12:**
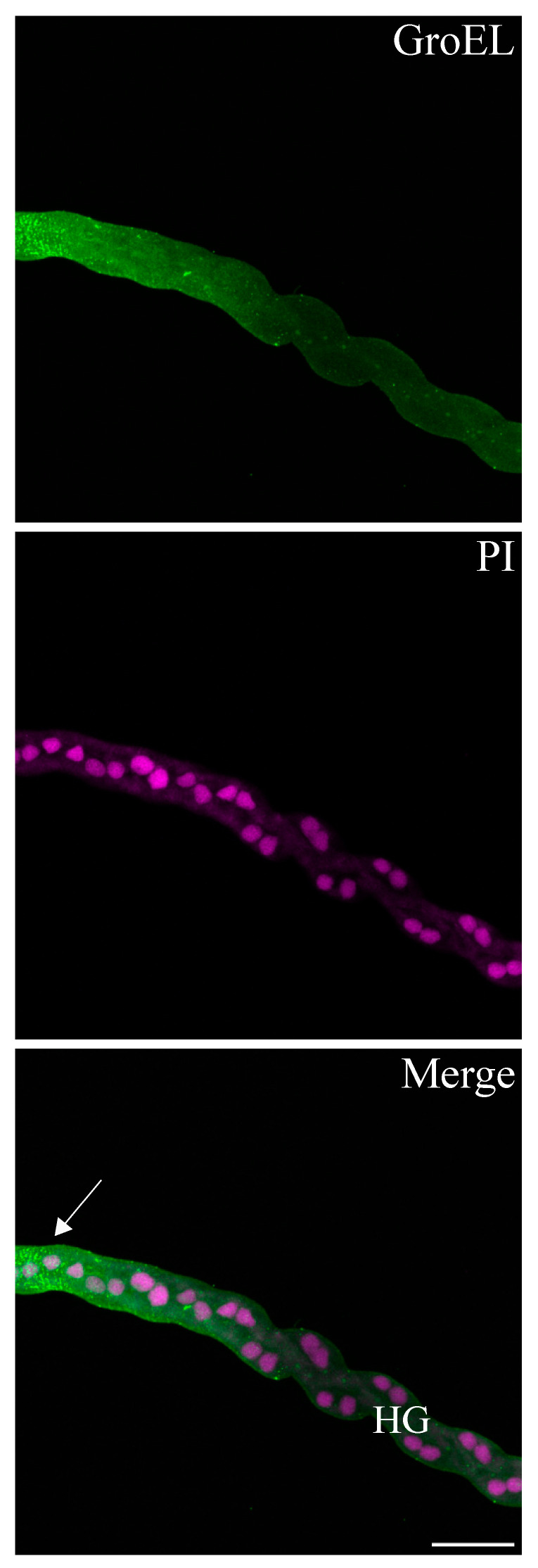
Immunofluorescence localization of GroEL using Alexa Fluor 488 (green) and cell nuclei were stained with propidium iodide PI (magenta) in the hindgut of the beet leafhopper. Images show minimal accumulation of GroEl within the hindgut (HG) cells, indicated by the weak signal of Alexa Fluor (arrow). Scale bar = 50 μM.

The GroEL signal was abundant in the leafhopper salivary gland lobes and associated parts (the stylet and salivary canal) (Figure 6, Figure 7 and Figure 8), the filter chamber (Figure 9). In the leafhoppers, the principal gland contains six types of cells; the largest and separated cells were type III, while the type VI cells situated at the center of the type V cells were the smallest. Immunofluorescence images showed that GroEl was mainly localized to type II and type III cells (Figure 7 and Figure 8). GroEL was detected in the muscles of the anterior midgut and the mid midgut (Figure 10), the Malpighian tubule (Figure 11), and in a few hindgut cells (Figure 12).

## 4. Discussion

Our research illustrates the unique localization of BCTV, a ssDNA virus (*curtovirus*, *geminiviridae*), in different organs within the beet leafhopper vector. The BCTV-CP was observed all over the midgut and mainly in the anterior midgut (Figure 1), suggesting that the virus particles are transferred through the cytoplasm of the filter chamber epithelial cells to the basal lamina to the midgut. This observation is consistent with a study that investigated the localization of a ssDNA virus (TYLCV) (*begomovirus*, *geminiviridae*), which was acquired by the whitefly (*Bemisia tabaci*) [27,28].

While an insect vector feeds on plant hosts, the virus particles are ejected into the plant phloem with vector saliva. Thus, the salivary glands of insect vectors serve as the last barrier to the circulation of the virus particles and, therefore, determine if the insect can transmit the pathogen successfully [29]. We found that the BCTV particles were distributed within the cell periphery in the PGs. However, our images showed a weak signal for BCTV in the salivary gland, which may refer to two reasons: First, the BCTV does not replicate in the vector, and for best results, a high ratio of viruliferous insects among the total population is necessary. Second, the primary antibody of BCTV-CP was synthesized through fusion protein technology, which may have reduced the antibodies’ effectiveness.

In this study, the immunolabeled GroEL protein was detected in high relative distribution in the salivary glands and filter chamber of the beet leafhopper vector (Figure 6, Figure 7, Figure 8 and Figure 9). This suggests that the locations where both BCTV and GroEL are present may also allow the virus to avoid the proteolytic environment of the hemocoel. Further studies need to confirm an in vivo interaction between BCTV-CP and the GroEL encoded by a primary endosymbiont in the C. tenellus vector. Morin et al. studied the interaction of GroEL-TYLCV in 1999. Using an overlay assay, they confirmed the interaction between GroEL synthesized by a secondary endosymbiont in *B. tabaci* and geminivirus tomato yellow leaf curl virus (TYLCV) particles [17]. Moreover, the coat protein of TYLCV and GroEL of *B. tabaci* interacted physically in the yeast two-hybrid assay [18].

To better understand the underlying mechanism of virus–vector interactions and transmission in ssDNA plant viruses (*geminiviridae* family), the localization of viruses within different genera in this family should be investigated. Previous studies showed that the circulative pathway of TYLCV (begomoviruses, *geminiviridae*) in the whitefly vector (*Bemisia tabaci*) [9] starts when the insect acquires the virus while feeding on plant phloem sap; the virus particles with other phloem components pass through the food canal in the insect stylet until reaching the esophagus. Since the esophagus is lined with chitin, it is not penetrated by virions [30]. The filter chamber (a modification of the digestive system) is the first tissue through which the virions can pass to the hemocoel. Based on extensive studies on TYLCV localization using fluorescence in situ hybridization (FISH), immunofluorescence, and transmission electron microscopy, the majority of TYLCV virions have been observed in the filter chamber, and the virus concentration decreased in other regions of the midgut. This suggests that it is the main site for viral particle translocation to the hemocoel [27,28].

In contrast to the family *Luteoviridae*, TYLCV is localized in midgut epithelial cells but not in the hindgut [7,27]. Once released from midgut cells into the hemocoel, they ultimately enter the salivary gland system, from which they can be transmitted successfully to new plants during feeding [6,9,30,31]. The vector specificity of geminiviruses is thought to be regulated at the gut interface [18,32] and by cells around the secretory region of the primary salivary glands [33].

The CP is the only known structural protein of geminiviruses [11]. Several researchers thoroughly investigated the role of the geminivirus CP in viral transmission; they conclude that the geminivirus CP is the major and possibly the sole determinant of the viral cycle within the insect, including virus passage through the midguts and ovary [8,10,34]. However, other insect vector proteins are thought to be involved in the viral transmission pathway. Due to a poorly understood mechanism, viruses are proposed to move in the hemolymph to the last cellular barrier, the salivary tissues, where they are suggested to enter the cells through endocytosis. Thus, they are successfully inoculated into new plant hosts during feeding [35].

To date, few reports have clarified the localization and interaction of geminiviruses within their vectors; an important follow-up question is whether these groups of viruses use a similar mechanism for the invasion of insect vectors. This study provides further evidence to understand the localization of a curtovirus within its vector. These and other investigations of pathogen–vector relationships are important to better understand how viruses behave in their vectors. Thus, novel ways can be found to combat such economically important plant disease organisms. Future work on other insect vector proteins could provide a clearer understanding of the role of this protein in mediating viral transmission.

## Data Availability

All data available from first author.
